# Latent profiles of college students’ AI literacy and their association with AI acceptance: the sequential indirect role of perceived affordances and growth mindset

**DOI:** 10.3389/fpsyg.2026.1926324

**Published:** 2026-08-19

**Authors:** Chuyuan Zhao, Tianxing Liu, Bingxin Gao

**Affiliations:** 1 School of Physical Education and Sport, Henan University, Kaifeng, China; 2 Physical Education Department, Dalian Minzu University, Dalian, China

**Keywords:** AI acceptance, AI literacy, chain mediation, growth mindset, latent profile analysis, perceived affordances

## Abstract

**Objective:**

Against the backdrop of the rapid proliferation of generative artificial intelligence (AI), fostering active acceptance and effective utilization of such technologies among college students has emerged as a critical concern in higher education. This study aims to investigate the relationship between AI literacy (AIL) and college students’ AI acceptance (AIA), as well as its underlying mechanisms, while employing latent profile analysis (LPA) to identify heterogeneous types of AIL and examine path differences across distinct latent classes.

**Methods:**

A sample of 975 Chinese college students completed the AIL Scale, Perceived Affordances (PA) Scale, Growth Mindset (GM) Scale, and AIA Scale.

**Results:**

AIL exhibited significant positive correlations with PA (*r* = 0.508, *p* < 0.001), GM (*r* = 0.458, *p* < 0.001), and AIA (*r* = 0.633, *p* < 0.001). Mediation analysis revealed that the indirect path via PA accounted for 6.26% of the total association, that of GM for 16.63%, and the chain mediating effect for 4.75%. LPA identified three distinct AIL profiles: low AIL–moderate ethics (26.87%), moderate AIL–low ethics (15.28%), and high-level balanced (57.85%). Notably, the mediating pathways differed across these latent classes, with significant differences observed primarily between the high-level balanced and moderate AIL–low ethics profiles.

**Conclusion:**

AIL was positively associated with college students’ AIA, and this association was partially accounted for by independent and sequential indirect paths through PA and GM. Moreover, AIL demonstrates a marked heterogeneous structure, with distinct latent classes exhibiting divergent patterns in path analysis. These findings provide empirical evidence for applying affordance theory and implicit theories of intelligence to the context of human AI collaboration, and suggest potential implications for differentiated AI education and mindset interventions in higher education settings.

## 1 Introduction

In the context of the rapid development of artificial intelligence (AI), large language models represented by ChatGPT, Deep Seek, and Gemini are profoundly reshaping college students’ learning modes and pathways of knowledge acquisition (Zhang, 2026). Therefore, the ability to effectively interact with artificial intelligence has become a crucial competence (Schiavo et al., 2024). However, the pace of transformation brought about by AI is so rapid that college students often find it difficult to adapt to and accept it promptly, and instead develop distrust and concerns regarding its rapid advancement. These concerns are specifically reflected in issues such as ethical controversies, information accuracy, privacy leakage, and employment impacts (Stahl and Wright, 2018; Türk et al., 2025). According to the *Global Student Wellbeing Report* 2025 jointly released by YouGov and Studiosity, a survey covering more than 10,000 college students across eight countries found that 79% of students have used AI tools in their learning, while 68% reported feeling pressured as a result, and a considerable proportion of students expressed concerns about their learning outcomes and confidence after using AI (Isabelle, 2025). Another global survey conducted by Chegg (covering 15 countries and nearly 12,000 college students) found that 80% of college students use generative AI to support their learning, while 53% expressed concerns about the accuracy of information (Organization, 2025). These findings indicate that, although AI demonstrates substantial application value across various domains, the aforementioned issues still largely constrain college students’ AI acceptance (AIA) (Ge et al., 2025). AIA refers to individuals’ behavioral intentions or willingness toward AI technologies, specifically manifested in their tendencies to use, purchase, or try related products or services (Venkatesh et al., 2003; Schiavo et al., 2024). It is noteworthy that the global survey conducted by Chegg also found that 69% of college students expect AI tools to be formally integrated into course instruction (Organization, 2025). This, to some extent, suggests that students do not fundamentally reject AI, but rather lack comprehensive understanding and systematic guidance for the effective use of AI technologies (Otto et al., 2024). Therefore, how to help college students develop a comprehensive and accurate understanding of AI and guide them to embrace AI development with a positive attitude has become an important issue that urgently needs to be addressed in higher education (Mukhamedkarimova and Umurkulova, 2025; Zhou and Xu, 2025).

To address this dilemma, increasing scholarly attention has been directed toward AIL as a key factor associated with individuals’ technology acceptance (Schiavo et al., 2024; Yim and Wegerif, 2024). From the perspective of perceived affordances (PA), only those affordances that are genuinely perceived by users are associated with their behavior (Volkoff and Strong, 2013; Lu et al., 2025). The cultivation of AI literacy (AIL) helps college students develop a deeper understanding of the practical value of AI tools such as ChatGPT, DeepSeek, and Gemini, enabling them to perceive the multiple functions of these tools in areas such as personalized learning and independent thinking. This, in turn, encourages them to more actively utilize these tools in their learning activities and significantly enhances their willingness to accept them (Li, 2023; Zhou and Peng, 2025). At the same time, implicit theory of intelligence provides another important perspective for understanding the above mechanisms. This theory posits that individuals’ implicit beliefs about whether intelligence or abilities are malleable profoundly influence their motivation and behavior, among which the incremental theory holds that abilities can be developed through effort (Dweck, 2006). Individuals with higher levels of AIL are more likely to believe that they can effectively master and utilize AI, possess a clear understanding of its advantages and limitations, and thus adopt a more positive and open attitude toward accepting and exploring AI technologies (Kaya et al., 2024; Schiavo et al., 2024). Furthermore, the study by Huang et al. provides direct evidence supporting PA as an antecedent variable of growth mindset (GM) (Huang et al., 2025). Their findings indicate that college students’ perceived usefulness of generative AI chatbots can significantly and positively predict GM, suggesting that when college students perceive AI tools as easy to use and genuinely helpful for improving learning outcomes, their belief in the malleability of their own abilities is correspondingly strengthened (Huang et al., 2025). Although existing indirect evidence suggests that AIL may positively influence AIA through pathways such as enhancing PA and GM, systematic research that comprehensively examines the relationships among these variables remains relatively scarce, with only a limited number of studies addressing some of these pathways indirectly (Li, 2023; Kaya et al., 2024; Schiavo et al., 2024; Yim and Wegerif, 2024; Huang et al., 2025; Türk et al., 2025; Zhou and Peng, 2025). Meanwhile, previous studies have predominantly explored the relationship between individuals and AIA based on technology acceptance models, with relatively few studies systematically examining the underlying mechanisms from the integrated perspectives of affordance theory and implicit theory of intelligence (Sánchez Prieto et al., 2020; Kelly et al., 2023; Pan et al., 2024). In addition, AIL itself has a multidimensional structure, encompassing core components such as knowledge, skills, values, and ethical awareness (Zhou et al., 2024). Influenced by differences in individual developmental experiences, college students may exhibit diverse configuration patterns across these dimensions (Zhang and Kang, 2026). If only average levels at the group level are assessed, the heterogeneity within the population may be easily obscured, making it difficult to effectively identify differences among various AIL profiles. The absence of such a research perspective may not only hinder the development of personalized intervention strategies but also lead to mismatches or underutilization of educational resources. In recent years, although several studies have identified heterogeneous categories of AI literacy among college students, none have examined whether the mediating pathways differ across these categories (Zhang and Kang, 2026; Zhu et al., 2026). Among the few studies that have combined latent profile analysis with mediation analysis, Ma et al. focused on innovative behavior, with AI anxiety and cognitive flexibility as mediators (Ma et al., 2026). Li et al. (2026) examined the relationship between AI literacy profiles and creativity, with information cocoons as the mediator. While these studies have advanced the understanding of AI literacy from different perspectives, no research to date has adopted an integrated framework of affordance theory and implicit theory of intelligence, with AI acceptance as the core outcome variable, to simultaneously identify heterogeneous AI literacy profiles and test whether the chain mediating pathways differ across these profiles.

To address the above challenges, this study integrates affordance theory and implicit theory of intelligence to construct a chain mediation model, aiming to reveal how AIL influences college students’ AIA through the mediating roles of PA and GM. At the same time, latent profile analysis is employed to identify the latent classes of AIL among college students, in order to uncover the heterogeneous structure within the population. This research design not only contributes to a deeper understanding of the psychological mechanisms through which AIL affects AIA, but also provides new insights into examining the diverse patterns of AIL and their relationships with AIA from the perspective of individual differences.

## 2 Literature review

### 2.1 AI literacy and AI acceptance

AIL refers to an individual’s ability to understand, use, and evaluate AI technologies, as well as to possess critical awareness of their social and ethical implications (Ji et al., 2025). As a multidimensional construct, AIL typically encompasses core elements such as knowledge, skills, ethics, and values (Zhou et al., 2024). In the context of rapid AI development, it has become a crucial digital literacy that everyone needs to acquire (Gerlich, 2025). Based on this, previous research has indicated that AIL is a key variable associated with college students’ AIA (Ke et al., 2025; Sun et al., 2026). On the one hand, individuals with lower AIL often lack sufficient understanding of the operational principles and potential risks of AI technologies, which over time tends to breed uncertainty and distrust, thereby fostering resistance toward AI (Wang et al., 2025). On the other hand, research has demonstrated that as individuals’ AIL continues to deepen, their knowledge reserves and technical application capabilities in the AI domain undergo substantive enhancement, leading to greater confidence in the reliability of outputs generated by AI tools (Bewersdorff et al., 2025a). Such elevated confidence helps alleviate technological anxiety during AI usage, strengthens self-efficacy, and consequently facilitates the formation of positive AI usage attitudes and usage intentions (Li H. et al., 2026). Furthermore, concerns regarding issues such as employment displacement, privacy breaches, and algorithmic deception posed by AI constitute significant affective barriers to AIA (Kai et al., 2026). Notably, Obadă et al. have pointed out that this issue can be effectively mitigated through systematic AIL education (Obadă et al., 2026). Specifically, systematic AIL education—particularly components encompassing ethical and value judgment competencies—enables individuals to adopt a more dialectical perspective on the risks and opportunities presented by AI, thereby facilitating rational acceptance of AI rather than blind rejection or excessive anxiety (Ng et al., 2024; Polat, 2025). Collectively, these findings converge upon a fundamental trend: as individuals’ understanding of artificial intelligence deepens, their AIA correspondingly exhibits an upward trajectory. Therefore, this study proposes:

*Hypothesis 1 (H1)*: AIL is positively associated with AIA.

### 2.2 The mediating role of PA

The concept of affordance was first proposed by Bornstein and Gibson (1980). He argued that affordance refers to the various action possibilities or functional opportunities that the environment or objects provide to individuals. PA is a core concept of affordance theory, developed by Norman based on Gibson’s theory of affordance (Norman, 1988). Applied to the field of artificial intelligence, PA refers to the action possibilities that individuals perceive during their interaction with AI systems, which can be utilized to accomplish specific tasks or satisfy particular needs (Wang et al., 2024; Zhang and Kang, 2026). It is noteworthy that affordance is not an inherent attribute of AI technology itself, but rather emerges from the interaction between users and technology—only those possibilities that are perceived by users can truly influence their usage intentions and behaviors (Zhang et al., 2024). Previous studies have found that individuals’ perceptions of the usefulness and ease of use of AI also significantly affect their level of acceptance (Davis, 1989; Cengiz and Peker, 2025). Specifically, when college students perceive AI tools as having high practical value and being able to provide personalized learning, as well as enhance learning interest and interactivity, their acceptance intention is usually higher (Li, 2023). Furthermore, when college students perceive that AI tools have low usage thresholds and intuitive operations, their level of trust in AI and their willingness to accept it both increase accordingly (Shin, 2023; Wang and Wang, 2024). Importantly, improvements in AIL can enhance individuals’ PA of AI (Lu et al., 2025). On the one hand, college students with higher levels of AIL are more capable of sensitively perceiving the deeper functions of AI in information integration, cross-domain knowledge linkage, and creative generation, thereby becoming more willing to regard it as a collaborator in the learning process (Chen and Xiao, 2025). On the other hand, as college students’ technical competence in using AI improves, the perceived usage threshold correspondingly decreases, making it easier for them to develop a sense of control and confidence in using AI, and thus become more willing to employ AI tools to solve real-world problems (Zhao et al., 2025). Accordingly, this study proposes:

*Hypothesis 2 (H2)*: PA plays a significant mediating role between AIL and college students’ AIA.

### 2.3 The mediating role of GM

GM refers to the belief that an individual’s abilities are not fixed, but can be developed and improved through sustained effort and effective learning strategies (Dweck, 2006). Existing studies have shown that AIL is significantly and positively correlated with individuals’ GM (Dönmez and Seferoğlu, 2025; Li H. et al., 2026). Specifically, the core value of AIL lies in stimulating individuals’ spirit of exploration and guiding them to continuously reflect on how to address real-world challenges with the help of AI technologies (Lin and Chen, 2024). From this perspective, this process essentially constitutes an effective training of GM (Yeager and Dweck, 2012; Li H. et al., 2026). In addition, college students with higher levels of AIL tend to possess stronger self-efficacy, and they are more confident in scientifically using AI tools to solve practical problems (Chen et al., 2026; Lin, 2026). In other words, as a comprehensive system of cognition and skills, AIL not only shapes college students’ ways of thinking in the AI era, but more importantly, it teaches them how to cope with challenges, set learning goals, and actively process feedback in the context of technological change—these capabilities are precisely the core manifestations of GM (Ng et al., 2024; Türk et al., 2025). Furthermore, GM is considered one of the key factors influencing AIA (Avcı, 2024). A study by Chow et al. confirmed that students who believe their AI-related abilities are malleable are more inclined to actively use AI tools (Chow and To, 2025). Meanwhile, individuals’ attitudes toward AI are critical determinants of their level of acceptance (Ismatullaev and Kim, 2024). Individuals with GM tend to adopt an open attitude toward the emergence of AI technologies, are able to sensitively identify the new opportunities embedded within them, and discover possibilities for self-improvement and innovative development (Weng, 2025). Accordingly, this study proposes:

*Hypothesis 3 (H3)*: GM plays a significant mediating role between AIL and college students’ AIA.

### 2.4 The chain mediating role of PA and GM

**Figure 1:**
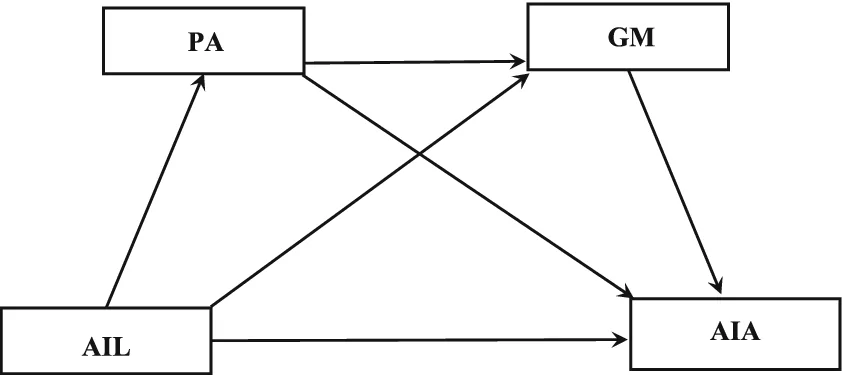
The proposed mediation model. AIL, AI literacy; PA, perceived affordances; GM, growth mindset; AIA, AI acceptance.

Previous studies have found that PA has a significant positive effect on the development of GM (Jang, 2023; Huang et al., 2025). Specifically, when individuals perceive AI tools as easy to use and effective in supporting learning, their belief in the malleability of their own abilities is correspondingly strengthened (Chen et al., 2024). Secondly, during interactions with AI, AI, as a conversational partner, can provide timely, specific, and constructive feedback, enabling individuals to accumulate successful experiences through repeated human–AI interactions, and such accumulation of experience further promotes the development of GM (Quan, 2025). In addition, Yeager et al. argued that mindsets are gradually constructed through accumulated cognitive experiences and feedback patterns (Yeager and Dweck, 2012). It follows that the formation of a stable belief logically requires prior perceptual experiences as its cognitive foundation. In other words, individuals must first perceive what AI can do for them; these repeated and consistent perceptual experiences are then gradually internalized into the belief that their AI-related competencies can be developed (Huang et al., 2025). On this basis, the present study posits that PA serves as the cognitive prerequisite for GM formation. Moreover, the evidence reviewed above provides solid empirical and theoretical support for perceived affordances as an antecedent variable of growth mindset. Further studies have found that when college students’ AIL is significantly improved, they are able to more sensitively perceive the affordances provided by AI technologies, thereby more effectively utilizing AI to solve real-world problems and promoting the development of their GM (Chen and Xiao, 2025). Meanwhile, existing research has shown that students with GM exhibit higher levels of AIA (Chow and To, 2025). Specifically, individuals with stronger GM are more likely to regard the emergence of AI as an opportunity for learning and growth, and to cope with challenges encountered during use with a more positive mindset, thereby more actively accepting AI technologies (Nunes et al., 2025). On the other hand, individuals who experience anxiety toward AI often display evident hesitation, avoidance, or even resistance when using related tools in practice (Taş and Turanlıgil, 2020). However, studies have shown that GM can significantly alleviate individuals’ levels of AI-related anxiety (Chow and To, 2025). This is because individuals with GM believe that their abilities can be continuously improved through learning and practice, and therefore are more inclined to view the rise of AI and the challenges encountered in its use as opportunities for capability development rather than threats to self-worth (Dinh, 2024). This positive cognitive appraisal effectively reduces anxiety toward AI technologies, thereby enhancing individuals’ willingness to use AI (Allan et al., 2022). Accordingly, this study proposes:

*Hypothesis 4 (H4)*: PA and GM play a significant chain mediating role between AIL and college students’ AIA (see Figure 1).

### 2.5 Potential structural differences in AIL

AIL of college students constitutes a composite capability that encompasses key dimensions such as knowledge reserves, skill application, value judgment, and ethical awareness, and is of critical significance for their sustainable development in the era of AI (Ji et al., 2025). Influenced by factors such as individual developmental experiences, educational background, and psychological traits, college students may exhibit diverse configuration patterns across these dimensions (Li X. et al., 2026; Wang et al., 2026). Identifying latent subgroups of AIL among college students therefore holds both important theoretical value and practical significance (Medina-Gual and Parejo, 2025; Wang et al., 2026). Theoretically, this research approach can effectively reveal the heterogeneity within the population and deepen the understanding of the structure and formation mechanisms of AIL. Practically, it can provide a more operational theoretical basis for educators to implement targeted instruction and personalized interventions, and offer important implications for enhancing college students’ AIA (Zhang and Kang, 2026; Zhu et al., 2026). At present, although most studies have explored the relationship between AIL and college students’ AIA, they are primarily based on a variable-centered perspective, and few studies have employed latent profile analysis to identify the latent profiles of AIL and examine their relationship with college students’ AIA (Schiavo et al., 2024; Ke et al., 2025). This study intends to adopt latent profile analysis to identify the latent profile structure of AIL among college students, and to examine the relationship between such profile structures and AIA, as well as differences in mediating pathways. Latent profile analysis is a person-centered statistical approach that classifies heterogeneous groups and estimates parameters based on individuals’ score patterns across multiple dimensions of AIL, thereby effectively avoiding issues associated with traditional methods, such as subjectively defined categories and substantial within-class heterogeneity (Yin et al., 2020; Zhu et al., 2026). Accordingly, this study proposes:

*Hypothesis 5 (H5)*: The pattern of indirect associations between AIL and AIA through PA and GM differs across latent subgroups.

## 3 Methods

### 3.1 Participants

**Figure 2:**
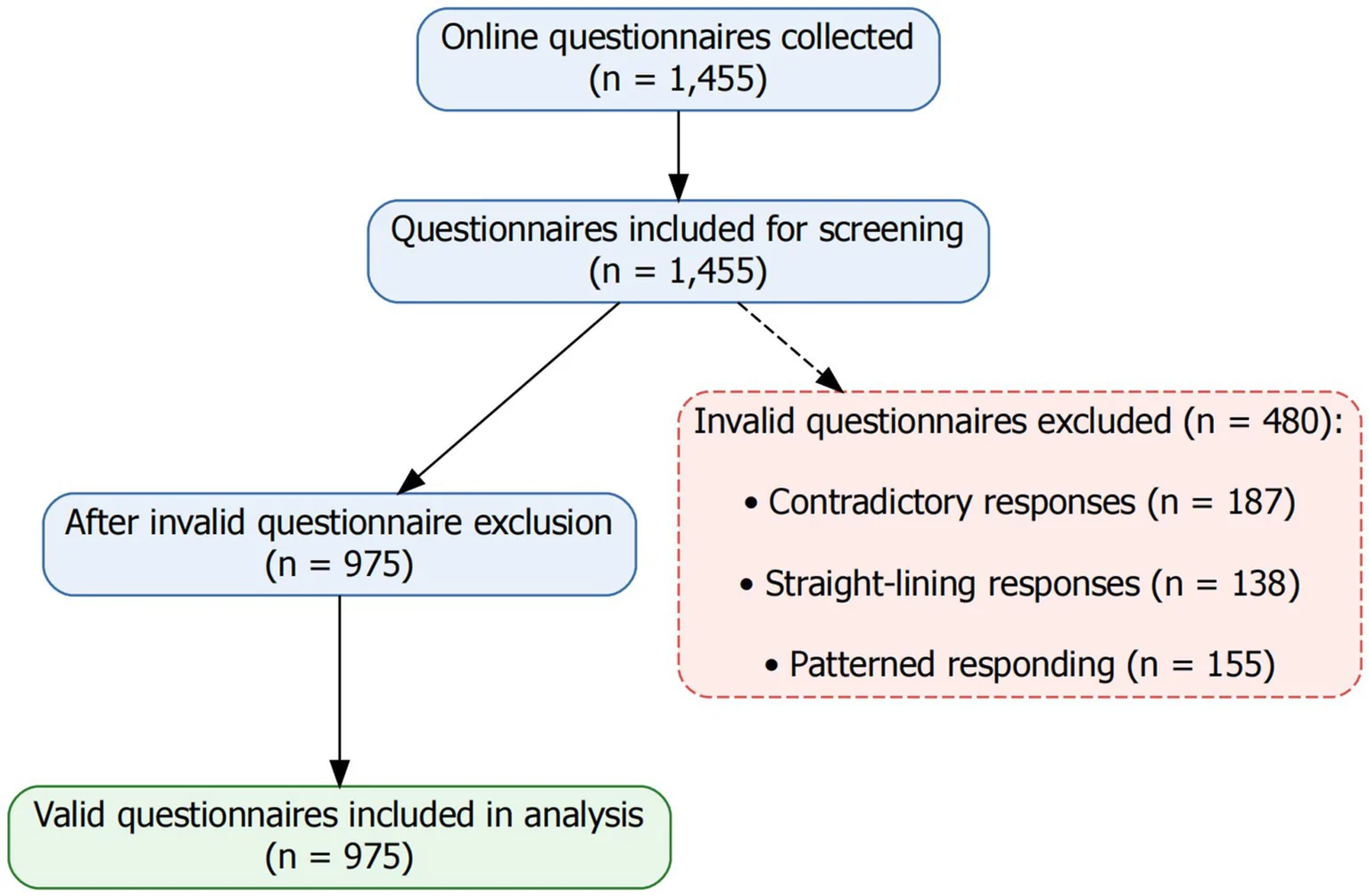
Steps in the screening process for research samples.

This study adopted a convenience sampling method and collected data through a professional online survey platform (Wenjuanxing, www.sojump.com). The sample consisted of college students from universities in Henan, Shandong, and Liaoning provinces in China, all of whom were at least 18 years old. Prior to participation, all respondents were fully informed of the research purpose, the anonymity of their responses, and their right to withdraw at any time. Each participant provided electronic informed consent. The study protocol was approved by the Biomedical Research Ethics Committee of Henan University. A total of 1,455 online questionnaires were distributed and returned. To ensure data quality, the online survey platform was configured with a minimum completion time of 90 s; submissions falling below this threshold were automatically rejected. During data screening, the remaining questionnaires were further examined and excluded based on the following criteria: (a) contradictory responses (e.g., endorsing both extreme low and extreme high ratings on conceptually similar items, *n* = 187); (b) invariant responses (e.g., selecting the same response option for over 70% of all items in the questionnaire, *n* = 138); and (c) patterned responses (e.g., alternating between two options in a predictable sequence, *n* = 155). After applying these exclusion criteria, 975 valid questionnaires were retained for subsequent analysis, yielding an effective response rate of 67.0% (see Figure 2). The final sample consisted of 539 males (55.28%) and 436 females (44.72%), with a mean age of 21.32 ± 1.54 (see Table 1). In terms of academic year, 212 students (21.74%) were freshmen, 263 (26.97%) were sophomores, 362 (37.13%) were juniors, and 138 (14.15%) were seniors.

**Table 1:** Distribution of basic information (*n* = 975).

Demographic variables		Number	Proportion (%)
Age (*M ± SD*)	21.32 ± 1.54	975	100%
Gender	Male	539	55.28%
	Female	436	44.72%
Grade	Freshmen	212	21.74%
	Sophomores	263	26.97%
	Juniors	362	37.13%
	Seniors	138	14.15%

M, Mean; SD, Standard deviation.

### 3.2 Measures

*AIL Scale*: The AIL scale revised by Zhou et al. (2024). This scale consists of 25 items, covering four dimensions: knowledge, skills, values, and ethical awareness. A 7-point Likert scale was used, ranging from “completely inconsistent” to “completely consistent,” scored from 1 to 7, with higher scores indicating higher levels of AIL among college students. In this study, confirmatory factor analysis showed the following fit indices: RMSEA = 0.06, GFI = 0.91, NFI = 0.89, and CFI = 0.91, indicating good structural validity. The Cronbach’s *α* coefficient of the scale was 0.86 (see Appendix Tables 4, 5 for AVE, CR, and HTMT results).

*AIA Scale*: The generative AI acceptance scale developed by Yilmaz et al. was used to measure AIA (Yilmaz et al., 2024). This scale includes 20 items, covering four dimensions: performance expectancy, effort expectancy, facilitating conditions, and social influence. A 5-point Likert scale was adopted, with higher scores indicating higher levels of AIA among college students. In this study, confirmatory factor analysis showed the following fit indices: RMSEA = 0.05, GFI = 0.94, NFI = 0.91, and CFI = 0.93, indicating good structural validity. The Cronbach’s *α* coefficient of the scale was 0.88 (see Appendix Tables 3, 6 for AVE, CR, and HTMT results).

*GM Scale*: The GM scale revised by Jia et al. (2022). This scale consists of 20 items, including several reverse-scored items. A 4-point Likert scale was used, with higher scores indicating a stronger GM among college students. In this study, confirmatory factor analysis showed the following fit indices: RMSEA = 0.03, GFI = 0.98, NFI = 0.97, and CFI = 0.98, indicating good structural validity. The Cronbach’s α coefficient of the scale was 0.83 (see Appendix Table 2 for AVE and CR results).

*PA Scale*: The PA scale developed by Zhou and Wu (2025). This is a unidimensional scale consisting of 4 items. A 7-point Likert scale was used, with higher scores indicating stronger PA of AI among college students. In this study, confirmatory factor analysis showed the following fit indices: RMSEA = 0.14, GFI = 0.98, NFI = 0.97, SRMR = 0.034 and CFI = 0.97. The elevated RMSEA can be attributed to the scale’s brief structure (4 items) and unidimensionality, as RMSEA tends to be inflated in models with low degrees of freedom (Kenny et al., 2015). Accordingly, we additionally report the SRMR value (0.034), which is well below the 0.08 threshold. According to the two-index strategy proposed by Hu and Bentler (1999), a model can be considered well-fitting when CFI ≥ 0.95 and SRMR ≤ 0.08; the CFI (0.97) and SRMR (0.034) for this scale both satisfy these criteria. Furthermore, factor loadings, AVE, and CR are presented in Appendix Tables 1, 8. Standardized factor loadings ranged from 0.65 to 0.76, AVE was 0.50, and CR was 0.80, all meeting conventional thresholds. Taken together, these indicators suggest that the validity of the scale is acceptable, though the elevated RMSEA warrants cautious interpretation. Finally, the Cronbach’s α coefficient of the scale was 0.80.

Discriminant validity across the four constructs was assessed using Heterotrait-Monotrait Ratio (HTMT), with all values below the 0.85 threshold (see Appendix Table 7).

### 3.3 Statistical analysis

This study employed Microsoft Excel, SPSSAU, and Mplus 8.3 for data processing and statistical analysis. First, Microsoft Excel was used to preprocess the raw data. Invalid questionnaires, including those with patterned responses and significantly short response times, were excluded through logical consistency checks to ensure data integrity and reliability. Subsequently, the screened valid data were imported into the SPSSAU online statistical platform for the following analyses. First, reliability and validity tests were conducted for each scale, with indicators such as GFI, RMSEA, and Cronbach’s α coefficients calculated to evaluate their psychometric properties. Second, descriptive statistical analyses were performed for the main study variables, and Pearson correlation analysis was conducted to examine the relationships among variables. Third, Harman’s single-factor test was applied to diagnose common method bias and to assess whether the total variance explained by a single factor was within an acceptable range. Finally, based on the Bootstrap resampling method (5,000 resamples, 95% confidence interval [CI]), a chain mediation model was constructed to examine the mediating effects and path relationships of PA and GM between AIL and AIA. To further reveal the heterogeneous structure of AIL among college students, latent profile analysis (LPA) was conducted using Mplus 8.3. This person-centered analytical approach classifies individuals with similar behavioral characteristics into the same category based on statistical models, thereby identifying latent subgroups within the population. For model fit evaluation, multiple indices were comprehensively considered, including the Akaike Information Criterion (AIC), Bayesian Information Criterion (BIC), sample-size adjusted Bayesian Information Criterion (aBIC), entropy (Entropy), Lo–Mendell–Rubin likelihood ratio test (LMR), and Bootstrap likelihood ratio test (BLRT). Models with one to four classes were fitted, and the optimal classification solution was determined based on statistical fit indices and theoretical interpretability. After determining the latent profile structure, a multi-group chain mediation analysis was further conducted. Specifically, the latent profiles obtained from Mplus 8.3 were used as grouping variables, and the Bootstrap resampling method (5,000 resamples, 95%CI) was applied in SPSSAU to examine whether the chain mediation paths differed significantly across groups.

Prior to the main analyses, the distribution of the age variable was examined. Although the Kolmogorov–Smirnov and Shapiro–Wilk tests were significant (*p* < 0.001), the skewness (0.206) and kurtosis (−0.606) were within the acceptable range for approximate normality (skewness < |2|, kurtosis < |7|) (Hair et al., 2010). Thus, age was considered sufficiently close to normal for parametric analyses.

## 4 Results

### 4.1 Common method bias test

Common method bias was assessed using both Harman’s single-factor test and confirmatory factor analysis. Exploratory factor analysis indicated that 11 factors had eigenvalues greater than 1, and the first factor accounted for 22.191% of the total variance, below the commonly used 40% threshold. This result suggests that no single factor accounted for the majority of the variance. In addition, confirmatory factor analysis was conducted on the AIL, PA, GM, and AIA scales to further assess potential common method bias. The CFA results provided further support for the view that common method bias was not a major concern in the present study (see Appendix Table 11 for details). Nevertheless, because the data were collected through self-report measures at a single time point, common method bias cannot be fully ruled out.

### 4.2 Descriptive statistics and correlation analysis

Descriptive statistics and correlation analyses were conducted for AIL, PA, GM, and AIA. As shown in Table 2, significant correlations were observed among the four variables (*p* < 0.001), providing a prerequisite for testing the hypotheses of this study. Specifically, AIL was significantly and positively correlated with PA (*r* = 0.508, *p* < 0.001), GM (*r* = 0.458, *p* < 0.001), and AIA (*r* = 0.633, *p* < 0.001). PA was significantly and positively correlated with GM (*r* = 0.382, *p* < 0.001) and AIA (*r* = 0.423, *p* < 0.001). AIA was also significantly and positively correlated with GM (*r* = 0.534, *p* < 0.001). These results provide a prerequisite for testing the hypotheses of this study.

**Table 2:** M, SD, and correlations between the variables.

Variables	M	SD	AIL	AIA	GM	PA
AIL	4.980	0.896	1			
AIA	3.592	0.656	0.633***	1		
GM	2.651	0.576	0.458***	0.534***	1	
PA	5.005	1.402	0.508***	0.423***	0.382***	1

*P < 0.05, **P < 0.01, ***P < 0.001.

### 4.3 Chain mediation effect analysis

As shown in Table 3, AIL (*β* = 0.458, *p* < 0.001), PA (*β* = 0.077, *p* < 0.01), and GM (*β* = 0.294, *p* < 0.001) were each significantly and positively associated with AIA. In addition, AIL was significantly and positively associated with PA (*β* = 0.509, *p* < 0.001) and GM (*β* = 0.356, *p* < 0.001), and PA (*β* = 0.202, *p* < 0.001) was significantly and positively associated with GM. Therefore, H1 is supported.

**Table 3:** Hierarchical multiple linear regression analysis results.

Variables	PA		GM		AIA	
	*β*	*t*	*β*	*t*	*β*	*t*
Gender	−0.010	−0.358	0.031	1.118	−0.017	−0.738
Age	−0.041	−1.427	0.018	0.635	0.009	0.385
Grade	0.020	0.691	0.017	0.587	−0.008	−0.354
AIL	0.509	18.401***	0.356	10.947***	0.458	16.053***
PA			0.202	6.215***	0.077	2.821**
GM					0.294	11.058***
*R^2^*	0.260		0.242		0.481	
*∆R^2^*	0.257		0.238		0.477	
*F*	85.278***		61.763***		149.230***	

R^2^ = proportion of variance explained; ΔR^2^ = change in R^2^ relative to the previous step; F = F-statistic for the overall model. *P < 0.05 **P < 0.01 ***P < 0.001.

**Figure 3:**
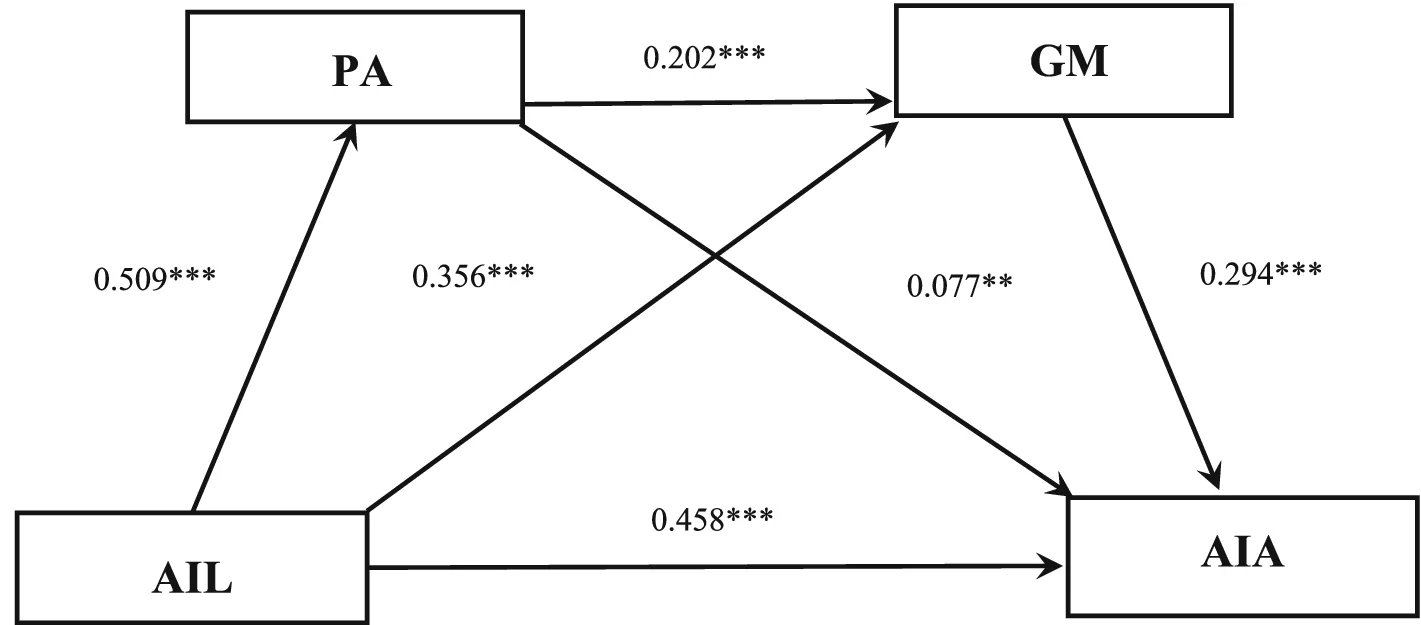
Chain mediation effects of PA and GM in the relationship between AIL and AIA.

Table 4 and Figure 3 indicate that among the three mediation pathways: (1) In Path 1, namely AIL → PA → AIA, the indirect effect of PA is 0.029, with a 95%CI [0.008, 0.050], accounting for 6.26% of the total effect; (2) In Path 2, namely AIL → GM → AIA, the indirect effect of GM is 0.077, with a 95%CI [0.057, 0.097], accounting for 16.63% of the total effect; (3) In Path 3, namely AIL → PA → GM → AIA, the indirect effect of PA and GM is 0.022, with a 95%CI [0.015, 0.031], accounting for 4.75% of the total effect. Therefore, H2, H3, and H4 are supported.

**Table 4:** Direct and indirect effects in the multiple mediator model.

Model	Effect	Boot SE	Boot LLCI	Boot ULCI	Relative mediation effect
Total effect	0.463	0.018	0.427	0.499	100%
Total direct effect	0.335	0.021	0.294	0.376	72.35%
Total indirect effect	0.128	0.014	0.101	0.155	27.65%
AIL ⇒ PA ⇒ AIA	0.029	0.011	0.008	0.050	6.26%
AIL ⇒ GM ⇒ AIA	0.077	0.010	0.057	0.097	16.63%
AIL ⇒ PA ⇒ GM ⇒ AIA	0.022	0.004	0.015	0.031	4.75%

Boot SE, Standard error of indirect effects; Boot LLCI, the lower bound of the 95% confidence interval; Boot ULCI, the upper limit of the 95% confidence interval (Percentile Bootstrap Method with Bias Correction).

### 4.4 Latent profile analysis of AIL

This study attempted to estimate latent class models of AIL with one to four profiles, and the fitting parameters of the four models were calculated and summarized (see Table 5). We tested one- to four-class models because the four-class solution already yielded a profile of only 62 individuals (6.4%), approaching the 5% minimum threshold for class stability (Yin et al., 2020). Testing five or more classes would almost certainly produce still smaller, unstable profiles. Among the latent profile model fit indices, lower values of AIC, BIC, and aBIC indicate better model fit; an Entropy value greater than 0.8 indicates that the classification accuracy exceeds 90%, and values closer to 1 represent better classification quality (Wang et al., 2017). In addition, when the *p* values of both the LMR and the BLRT are less than 0.05, the model with *K* classes is considered to be significantly better than the model with *K − 1* classes. Among the four profile models, the values of AIC, BIC, and aBIC decreased continuously as the number of latent profile classes increased. Moreover, the Entropy values of the two- to four-profile models were all greater than 0.8. The p values of LMR and BLRT for the two- and three-profile models both reached statistical significance (*p* < 0.05), whereas the LMR *p* value for the four-profile model was 0.39 (*p* > 0.05), which was not significant. Taken together, the two-class solution showed poorer fit than the three-class solution and was insufficient to fully capture the heterogeneity of college students’ AIL. The four-class solution offered marginally better fit but introduced a small profile of only 62 individuals (6.4%), approaching the 5% minimum class size threshold and raising concerns about solution reliability. The three-profile model thus achieved the best balance of statistical adequacy, classification robustness, and interpretability (Rindskopf, 2003). In addition, the stability of the three-profile solution was also confirmed (see Appendix Table 4 for details).

**Table 5:** Model fit indicators for different profiles of AIL.

Class	AIC	BIC	aBIC	Entropy	LMR(*P*)	BLRT(*P*)	Group Size	Class Probability
1	76475.248	76670.545	76543.505	–	–	–	975	-
2	73878.920	74176.749	73983.013	0.865	<0.001	<0.001	343/632	0.35/0.65
3	72929.873	73330.233	73069.800	0.888	<0.05	<0.001	262/149/564	0.27/0.15/0.58
4	72282.247	72785.138	72458.009	0.880	0.39	<0.001	62/487/274/152	0.06/0.50/0.28/0.16

### 4.5 Characteristics of AIL latent profiles

**Figure 4:**
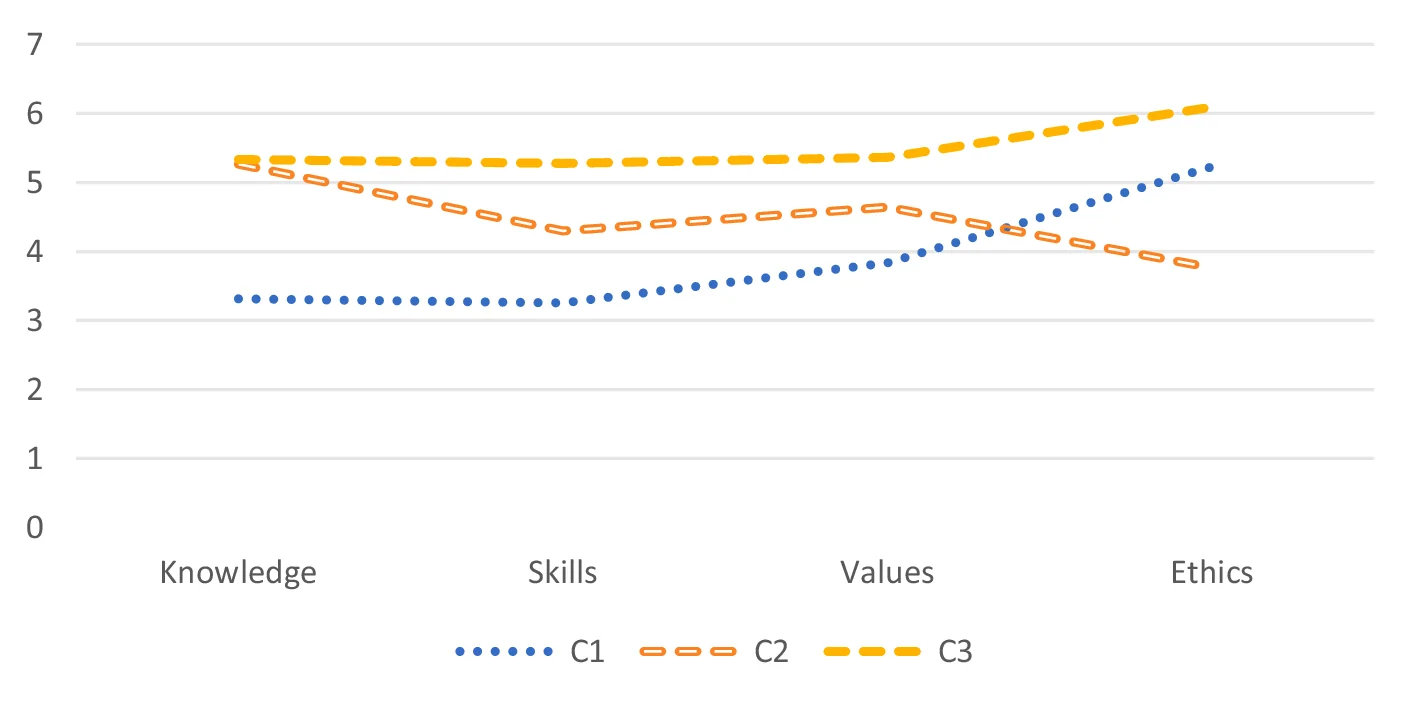
Estimated conditional means of latent classes for AIL. C1, low AIL–moderate ethics; C2, moderate AIL–low ethics; C3, high-level balanced.

The three profiles were labeled based on their mean scores on the four AIL dimensions relative to each other; the latent classes are presented in Figure 4. The estimated scores of the knowledge dimension in Class 1 (C1) were lower than those of the other groups, while the estimated scores of the ethical dimension were higher than those of Class 2 (C2) but lower than those of Class 3 (C3). This group included 262 individuals (26.87%) and was labeled as the “low AIL-moderate ethics” group. The scores of the knowledge dimension in C2 were higher than those of C1 and slightly lower than those of C3, whereas the scores of the ethical dimension were lower than those of the other groups. This group included 149 individuals (15.28%) and was labeled as the “moderate AIL-low ethics” group. The scores of C3 were higher than those of the other two groups across dimensions, including 564 individuals (57.85%), and was labeled as the “high-level balanced” group. It should be noted that the smallest profile (C2) comprised 149 individuals (15.28%), well above the 5% minimum threshold for class stability (Yin et al., 2020), ruling out the possibility of a spurious class. In addition, the three latent classes of college students’ AIL showed clear differences in mean scores on the AIL scale (see Table 6).

**Table 6:** Descriptive statistics and difference tests of AIL among the three groups (*M ± SD*).

Dimensions	Category			*F*	*Post Hoc* Test
	C1 (*n* = 262)	C2 (*n* = 149)	C3 (*n* = 564)		
Knowledge	3.291 ± 1.379	5.289 ± 1.396	5.332 ± 1.278	225.536***	C3 > C2 > C1
Skills	3.214 ± 1.038	4.329 ± 1.014	5.276 ± 0.898	422.155***	C3 > C2 > C1
Values	3.834 ± 1.324	4.617 ± 1.124	5.363 ± 0.972	176.492***	C3 > C2 > C1
Ethics	5.207 ± 0.958	3.742 ± 0.829	6.091 ± 0.649	571.312***	C3 > C1 > C2

*P < 0.05, **P < 0.01, ***P < 0.001.

### 4.6 The relationship between AIL latent profiles and AIA

To examine the relationship between AIL and college students’ AIA, a one-way analysis of variance (ANOVA) was conducted, with latent classes of AIL as the independent variable and the mean score of AIA as the dependent variable (see Table 7). The results indicated that there were significant differences in AIA among college students across different latent classes of AIL (*F* = 181.383, *p* < 0.001). *Post hoc* comparisons further revealed that the mean scores of AIA among college students in different latent classes of AIL, from highest to lowest, were as follows: C3 > C2 > C1.

**Table 7:** Descriptive statistics and difference tests of AIA among the three groups (*M ± SD*).

Variables	Category			*F*	*Post Hoc* Test
	C1 (*n* = 262)	C2 (*n* = 149)	C3 (*n* = 564)		
AIA	3.118 ± 0.641	3.345 ± 0.633	3.877 ± 0.495	181.383***	C3 > C2 > C1

*P < 0.05, **P < 0.01, ***P < 0.001.

### 4.7 Chain mediation analysis based on latent profile classes of AIL

To examine whether the mediating roles of PA and GM between AIL and AIA are influenced by different latent classes of AIL among college students, a multi-group chain mediation model was employed. The three latent classes were treated as grouping variables, with C2 as the reference group, to conduct the chain mediation analysis. The results (see Table 8) showed that, compared with C2, C1 was significantly negatively associated with GM (*β* = −0.126, *p* < 0.01) and AIA (*β* = −0.103, *p* < 0.01), while its association with PA (*β* = −0.036, *p* > 0.05) was not significant. Compared with C2, C3 was significantly positively associated with PA (*β* = 0.409, *p* < 0.001), GM (*β* = 0.226, *p* < 0.001), and AIA (*β* = 0.222, *p* < 0.001). In addition, PA was significantly positively associated with GM (*β* = 0.244, *p* < 0.001) and AIA (*β* = 0.162, *p* < 0.001), and GM was significantly positively associated with AIA (*β* = 0.341, *p* < 0.001).

**Table 8:** Hierarchical multiple linear regression analysis results (vs. C2).

Variables	PA		GM		AIA	
	*β*	*t*	*β*	*t*	*β*	*t*
Gender	−0.015	−0.501	0.027	0.962	−0.024	−0.987
Age	−0.034	−1.146	0.020	0.673	0.014	0.551
Grade	0.027	0.905	0.025	0.859	−0.003	−0.119
AIL (C1)	−0.036	−0.878	−0.126	−3.133**	−0.103	−2.905**
AIL (C3)	0.409	9.952***	0.226	5.372***	0.222	5.937***
PA			0.244	7.805***	0.162	5.732***
GM					0.341	12.057***
*R^2^*	0.191		0.235		0.410	
*∆R^2^*	0.187		0.231		0.406	
*F*	45.901***		49.645***		95.966***	

AIL (C1) and AIL (C3) are dummy-coded variables representing the comparison of C1 and C3, respectively, against the reference group C2. *P < 0.05 **P < 0.01 ***P < 0.001.

**Figure 5:**
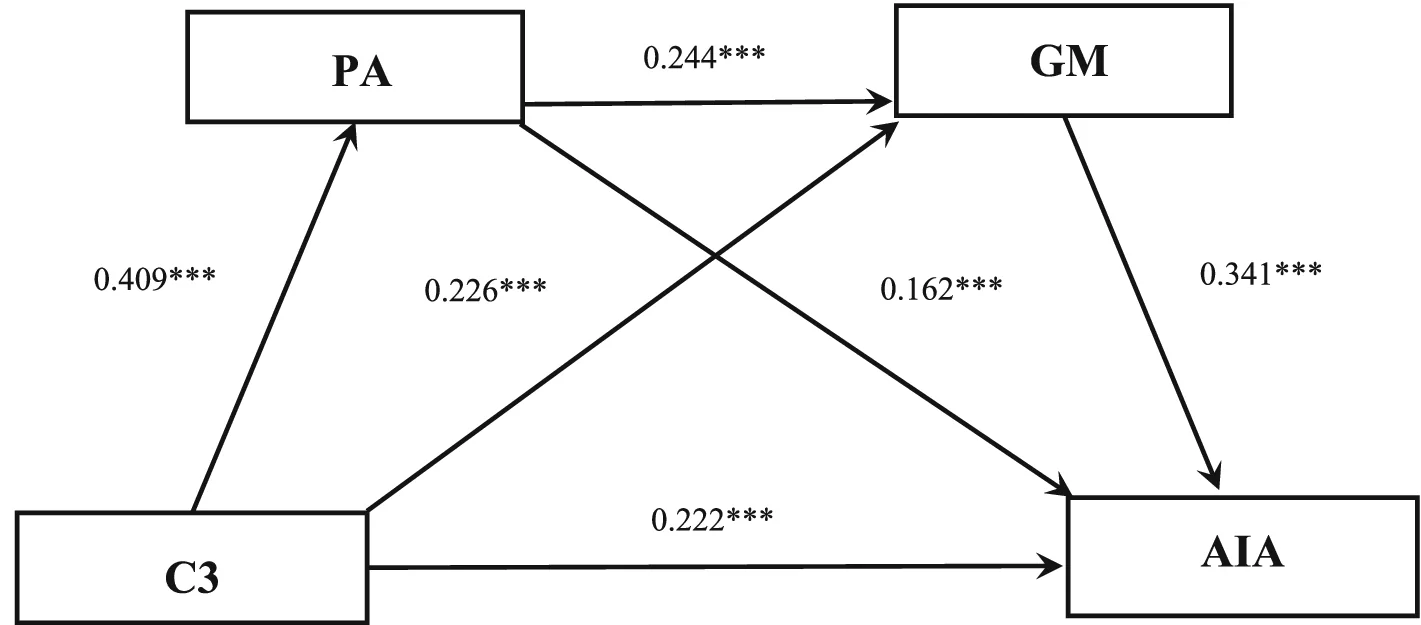
Chain mediation effects of PA and GM in the relationship between AIL and AIA (C3 vs. C2).

Table 9 and Figure 5 present the mediation effect analysis for C3 relative to C2. The results showed that among the three mediation pathways: (1) In Path 1, namely AIL → PA → AIA, the indirect effect of PA was 0.088, with a 95%CI [0.053, 0.126], accounting for 16.60% of the total effect; (2) In Path 2, namely AIL → GM → AIA, the indirect effect of GM was 0.102, with a 95%CI [0.063, 0.144], accounting for 19.25% of the total effect; (3) In Path 3, namely AIL → PA → GM → AIA, the indirect effect of PA and GM was 0.045, with a 95%CI [0.030, 0.063], accounting for 8.49% of the total effect.

**Table 9:** Direct and indirect effects in the multiple mediator model (C3 vs. C2).

Model	Effect	Boot SE	Boot LLCI	Boot ULCI	Relative mediation effect
Total effect	0.530	0.052	0.429	0.632	100%
Total direct effect	0.295	0.050	0.198	0.393	55.66%
Total indirect effect	0.235	0.030	0.179	0.294	44.34%
AIL ⇒ PA ⇒ AIA	0.088	0.019	0.053	0.126	16.60%
AIL ⇒ GM ⇒ AIA	0.102	0.020	0.063	0.144	19.25%
AIL ⇒ PA ⇒ GM ⇒ AIA	0.045	0.008	0.030	0.063	8.49%

**Figure 6:**
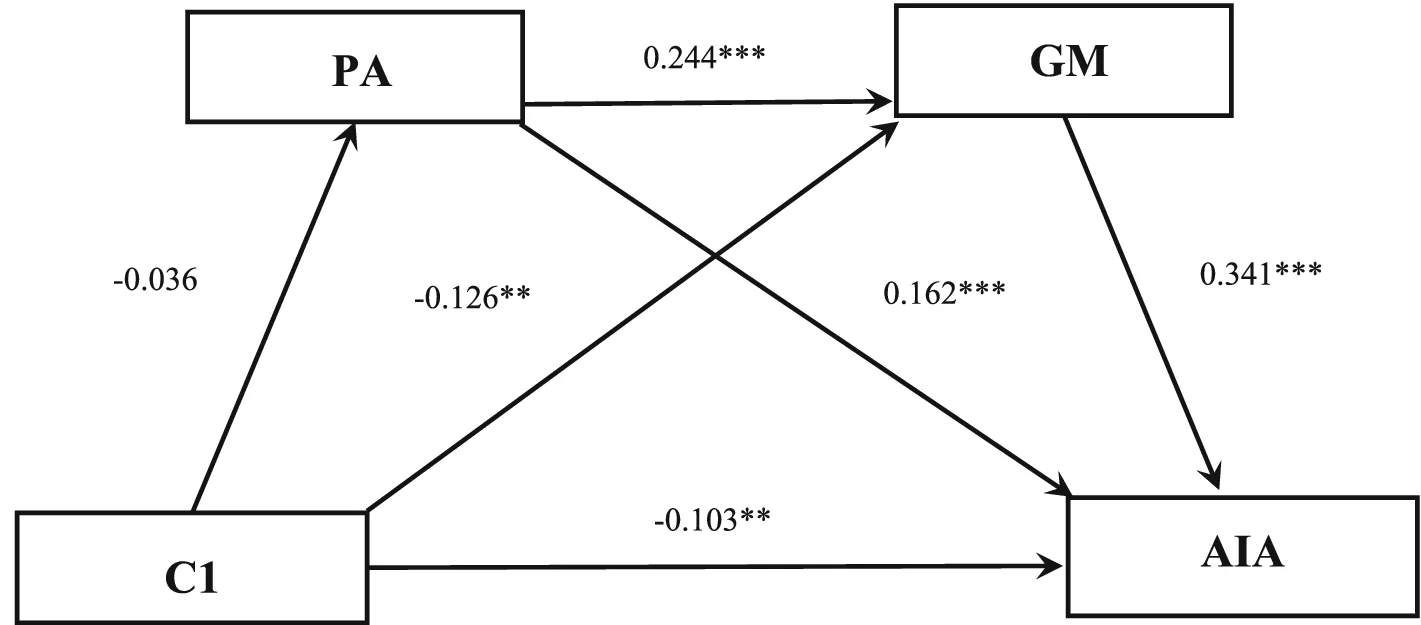
Chain mediation effects of PA and GM in the relationship between AIL and AIA (C1 vs. C2).

Table 10 and Figure 6 present the mediation effect analysis for C1 relative to C2. The results showed that among the three mediation pathways: (1) In Path 1, namely AIL → PA → AIA, the indirect effect of PA was −0.009, with a 95%CI [−0.031, 0.013], accounting for 3.95% of the total effect; (2) In Path 2, namely AIL → GM → AIA, the indirect effect of GM was −0.063, with a 95%CI [−0.106, −0.023], accounting for 27.63% of the total effect; (3) In Path 3, namely AIL → PA → GM → AIA, the indirect effect of PA and GM was −0.004, with a 95%CI [−0.016, 0.006], accounting for 1.75% of the total effect. Taken together, while all three mediation pathways showed significant differences between C3 and C2, the differences between C1 and C2 were significant only for the AIL → GM → AIA pathway, whereas the AIL → PA → AIA pathway and the chain mediation pathway did not reach significance. Thus, H5 is partially supported, as the C3 vs. C2 comparison revealed significant differences across all three mediating pathways, whereas the C1 vs. C2 comparison showed significant differences only in the AIL → GM → AIA pathway.

**Table 10:** Direct and indirect effects in the multiple mediator model (C1 vs. C2).

Model	Effect	Boot SE	Boot LLCI	Boot ULCI	Relative mediation effect
Total effect	−0.228	0.058	−0.342	−0.115	100%
Total direct effect	−0.152	0.052	−0.255	−0.049	66.67%
Total indirect effect	−0.076	0.027	−0.131	−0.022	33.34%
AIL ⇒ PA ⇒ AIA	−0.009	0.011	−0.031	0.013	3.95%
AIL ⇒ GM ⇒ AIA	−0.063	0.021	−0.106	−0.023	27.63%
AIL ⇒ PA ⇒ GM ⇒ AIA	−0.004	0.006	−0.016	0.006	1.75%

## 5 Discussion

This study systematically examined the relationship between AIL and AIA, as well as its underlying mechanisms. The results indicate that AIL was significantly and positively associated with AIA. After incorporating PA and GM as mediating variables, both the direct and indirect effects of AIL on AIA remained significant, suggesting that AIL was not only directly associated with AIA but also showed indirect associations through the independent and chain mediating pathways of PA and GM. It is noteworthy that the independent mediating effect of PA accounted for only 6.26% of the total effect, while the chain mediating effect of PA and GM accounted for only 4.75% of the total effect. Therefore, the interpretation of the findings of this study should remain cautious. In addition, this study employed LPA to identify the latent classes of AIL among college students, and the results classified them into three types: low AIL-moderate ethics, moderate AIL-low ethics, and high-level balanced. Further chain mediation analysis based on latent profiles revealed that there was no significant difference between C1 and C2, whereas a significant difference was observed between C3 and C2.

### 5.1 The direct effect of AIL on AIA

This study found that, after controlling for the mediating variables, the direct effect of AIL on AIA remained significant, thereby supporting H1. This finding is generally consistent with previous studies (Schiavo et al., 2024; Ke et al., 2025; Türk et al., 2025). Research has shown that, as college students’ knowledge, skills, and cognitive understanding of the field of artificial intelligence continue to deepen, their anxiety and concerns about AI tend to decrease. This, in turn, helps to reduce the negative avoidance tendencies caused by technological fear and enhances individuals’ level of acceptance of AI (Bewersdorff et al., 2025b). Therefore, improving college students’ AIA, transforming negative cognitive patterns, and cultivating and strengthening their AIL have become key intervention pathways (Cengiz and Peker, 2025). Hornberger et al. (2025) assessed the AIL of 1,465 university students across Germany, the UK, and the US and found that higher AIL was consistently associated with more positive AI attitudes. Similarly, in a Turkish sample, Türk et al. (2025) confirmed through a moderated mediation model that AIL positively predicted generative AI acceptance. These converging findings from distinct cultural contexts suggest that the positive association between AIL and AIA may extend beyond the Chinese educational setting. Specifically, improvements in AIL enable college students to more clearly perceive the functional advantages and inherent limitations of AI technologies, thereby prompting them to accept and apply AI tools in a more rational manner (Wang et al., 2024; Toker Gokce et al., 2025). For example, the enhancement of knowledge and skills dimensions provides solid technical and operational support for diverse task contexts, thereby effectively improving college students’ confidence and practical capabilities in information processing and complex problem-solving, which lays a solid cognitive and capability foundation for their acceptance and application of AI tools (Ng et al., 2024). The deepening of the ethical awareness dimension strengthens individuals’ trust in AI technologies and their willingness to accept them by mitigating cognitive biases and resolving potential ethical barriers (Zhang et al., 2025). In addition, the capacity for value judgment regarding AI helps to cultivate individuals’ positive affective orientation toward AI technologies, enabling them to more accurately perceive the usability and ease of use of AI in specific contexts (Sun et al., 2026). Through the construction of such positive cognitive schemas and the reinforcement of favorable usage expectations, individuals’ overall attitudes toward AI become more positive, ultimately promoting the enhancement of AIA among college students.

### 5.2 The mediating role of PA

This study further found that AIL was indirectly associated with college students’ AIA through PA, thereby supporting H2. This finding provides additional empirical support for affordance theory, suggesting that when individuals have clearer and richer PA of AI tools, they are more likely to recognize the value of AI and tend to accept and use it (Norman, 1988; Wang et al., 2024). The findings of Lu et al. also support this logic, indicating that AIL can deepen students’ understanding of AI functions, interactions, and ethical boundaries, reduce usage barriers, and thereby influence their AI usage behaviors (Lu et al., 2025). Notably, a study of 676 Saudi Arabian university students found that AIL was a strong predictor of perceived usefulness (Al-Abdullatif and Alsubaie, 2024). Similarly, through semi-structured interviews and observation with 23 students from the UK, China, the US, and other countries at a British university, students were found to perceive and utilize ChatGPT’s affordances for writing in highly individualized ways, suggesting the pervasiveness of AI affordance perception among students from diverse cultural backgrounds (Zhao et al., 2024). Collectively, these lines of evidence suggest that the mediating role of PA in the AI acceptance process may not be limited to the Chinese educational context. From the perspective of perceived usefulness, college students with higher levels of AIL are more likely to recognize the practical utility of AI in problem-solving, improving efficiency, and optimizing tasks, and such perceptions of usefulness significantly enhance their willingness to use AI (Lijie et al., 2025). From the perspective of perceived ease of use, students with higher AIL are able to interact effectively with AI, which reduces the difficulty they perceive in using AI (Ke et al., 2025; Liu et al., 2025). This positive perception of ease of use lowers psychological barriers to AI usage—for example, students no longer perceive AI tools as complex or difficult to operate—thereby increasing their tendency to try and continuously use AI (Shao et al., 2024). It is worth noting that the indirect effect via PA was modest, accounting for only 6.26% of the total effect. Accordingly, interpretations of this specific pathway should remain cautious.

### 5.3 The mediating role of GM

This study found that AIL was also indirectly associated with college students’ AIA through GM, thereby supporting H3. This finding further extends the explanatory boundary of implicit theory of intelligence in the domain of AIA. On the one hand, cultivating college students’ AIL helps them master the use of AI tools, thereby enabling them to effectively apply the acquired skills in practical tasks such as academic activities (Zhou et al., 2024). When students successfully complete tasks with the assistance of AI tools and obtain positive usage experiences, such experiences further enhance their confidence in their own AI-related abilities, leading them to believe that these abilities are malleable and can be continuously improved through learning and practice (Li H. et al., 2026). On the other hand, college students who lack AIL are more likely to perceive AI technologies as mysterious, complex, or even intimidating, which may induce avoidance tendencies toward AI tools (Zhang and Yu, 2025; Kaya and Keskin, 2026). Over time, such persistent avoidance behaviors may contribute to the formation of a fixed mindset (Li H. et al., 2026). In contrast, with the improvement of AIL, college students gradually deepen their understanding of AI. They not only recognize that proficient use of AI is attainable, but also clearly perceive its widespread applications and developmental potential across various domains, thereby stimulating their intrinsic motivation to learn and master AI-related skills and ultimately fostering GM (Liu et al., 2025; Li H. et al., 2026). Furthermore, college students with GM are more inclined to accept and use AI tools (Chow and To, 2025). For instance, Avcı found among Turkish university students that a growth creative mindset was one of the strongest predictors of GAI acceptance, a finding highly consistent with the present results (Avcı, 2024). In addition, a study by Bewersdorff et al. using a sample of German university students demonstrated that students who believe in their ability to learn and master AI tend to use AI tools more actively (Bewersdorff et al., 2025a). Specifically, such students are more likely to regard AI as an opportunity to enhance their own abilities rather than as a threat, and this positive attitude has been shown to be an important prerequisite for improving AIA (Avcı, 2024; Zhao et al., 2025). In addition, college students with GM are more willing to invest time and effort in exploring the potential uses of AI, enabling them to genuinely experience the benefits brought by AI, thereby strengthening their intention and actual behavior of adopting AI technologies (Chen and Yi, 2024).

### 5.4 The chain mediating role of PA and GM

This study further found that AIL was indirectly associated with college students’ AIA through the chain pathway of PA and GM, thereby supporting H4. This finding provides empirical evidence for integrating affordance theory and implicit theory of intelligence in the context of AI acceptance research, and suggests a potential PA and GM chain mechanism that warrants further investigation. Specifically, college students with higher levels of AIL are able to effectively perceive the affordances of AI tools, rather than viewing them as adversarial or worrying about being replaced by AI (Zhou and Wu, 2025). On this basis, when facing learning or task-related situations, college students are more willing to actively attempt to use AI tools for practical exploration (Zhang et al., 2025). At the same time, when college students genuinely experience the convenience and efficiency brought by AI technologies, they are more likely to attribute such positive experiences to the success of their own learning and adaptive capabilities. This, in turn, strengthens their belief that abilities can be developed through effort and learning, thereby enhancing their willingness to accept and use AI technologies (Avcı, 2024; Chow and To, 2025). Although the chain mediation effect was statistically significant, its proportion of the total effect was also limited (4.75%), indicating that the PA and GM chain pathway serves more as a supplementary mechanism than a dominant explanatory route. Future research may explore additional mediators to further elucidate the AIL and AIA relationship.

### 5.5 Path differences across latent classes of AIL

This study further found that there are three distinct latent classes of AIL among college students. The chain mediation analysis based on latent profiles revealed that there was no significant difference between C1 and C2, whereas a significant difference was observed between C3 and C2, thereby partially supporting H5. Specifically, compared with C2, college students in C3 performed better across all four dimensions of AIL. This indicates that they are more proficient in acquiring, communicating, and applying AI tools, possess stronger evaluative thinking and ethical awareness, and hold more positive value orientations toward AI (Zhu et al., 2026). These advantages further enrich college students’ PA of AI technologies, making them more inclined to regard AI as an effective tool for enhancing their own abilities and promoting self-development, thereby embracing the AI era with a more positive attitude (Lu et al., 2025). This further suggests that, compared with the single-dimension (knowledge dimension) prominence observed in C2, the comprehensive cultivation of AIL across multiple dimensions is essential for laying a solid foundation for college students’ sustainable development in the AI era. In contrast, although no significant differences were observed between C1 and C2 in the chain mediation pathways, the performance of C1 was markedly weaker than that of C2 in terms of the total effect, total direct effect, total indirect effect, and the AIL → GM → AIA pathway. Drawing on previous studies, it can be inferred that, compared with the single-dimension (ethical dimension) prominence of C1, the combined performance across the knowledge, skills, and value dimensions showed a stronger association with college students’ AIA and its related mediating pathways (Tripopsakul and Ratanavanich, 2026; Zhang and Kang, 2026). The findings of Zhu et al. also support this perspective, indicating that college students with generally low levels across all dimensions of AIL exhibit notable deficiencies in AI evaluation and usage (Zhu et al., 2026). Specifically, such students are unable to effectively assess the validity of AI-generated content and are prone to either uncritically accepting or rejecting AI recommendations in learning or task contexts, thereby forming inaccurate perceptions of AI technologies (Yan et al., 2025; Zhang, 2026). Meanwhile, these students are more likely to experience anxiety due to the uncertainty brought by AI technologies, which in turn inhibits the development of GM, causes them to miss opportunities for learning and development enabled by AI, and ultimately results in lower willingness to accept AI (Chakraburty et al., 2025). In summary, compared with AIL structures characterized by single-dimension prominence, a comprehensive AIL model featuring the coordinated development of knowledge, skills, and values is more conducive to enhancing PA of AI technologies, strengthening GM, and thereby promoting college students’ positive acceptance and sustainable use of AI.

### 5.6 Implications and limitations

Theoretical Implications: Based on affordance theory and implicit theory of intelligence, this study constructed and tested a chain mediation model in which AIL influences AIA through PA and GM, thereby revealing the underlying psychological mechanisms through which AIL affects college students’ AIA. First, the study confirmed the independent and chain mediating roles of PA and GM between AIL and AIA, extending the application boundary of affordance theory in human–AI interaction contexts and providing new empirical support for the applicability of implicit theory of intelligence in the field of technology acceptance. Second, the study employed latent profile analysis to identify three heterogeneous types of AIL among college students (low AIL–moderate ethics, moderate AIL–low ethics, and high-level balanced), and found that the chain mediation pathways differed across these groups in an asymmetric manner, with more pronounced differences between the high-level balanced and moderate AIL–low ethics profiles. This finding goes beyond the traditional variable-centered perspective and reveals the structural heterogeneity of AIL and its differential effects on AIA from a person-centered perspective, providing a theoretical basis for precision-oriented AI education interventions. In addition, the study found that the mediating effect of GM accounted for the largest proportion of the total effect (16.63%), suggesting that greater attention should be paid to the cultivation of students’ mindset in AI education. Practical Implications: The findings provide specific implications for AI education practices in higher education institutions. First, universities should systematically enhance students’ AIL, particularly in the dimensions of knowledge and skills, while also emphasizing the cultivation of values and ethical awareness, so as to strengthen students’ PA of AI technologies and promote more positive attitudes toward AIA. Second, given the important role of GM in the chain mediation model, educators can cultivate students’ GM by designing challenging tasks, providing constructive feedback, and emphasizing the value of effort and strategies, thereby enhancing college students’ willingness to use AI. Third, based on the three student groups identified through LPA, educators should implement differentiated intervention strategies. Specifically, for the low AIL–moderate ethics group, foundational skill training and psychological guidance should be provided to help them build basic understanding and confidence in using AI; for the moderate AIL–low ethics group, practical training and ethical education should be strengthened to promote a transition from “knowing how to use” to “using appropriately”; for the high-level balanced group, advanced courses and innovative project opportunities should be offered to enable them to play leading and exemplary roles. Such precision-oriented educational strategies can improve the efficiency of educational resource allocation and promote equitable benefits from AI technological development for all college students.

Despite the theoretical and practical contributions of this study, several limitations should be acknowledged. First, this study adopted a cross-sectional survey design, with all variables collected at a single time point, which precludes causal inferences. Future studies may employ longitudinal designs or experimental interventions to more rigorously test causal relationships among variables. Second, the sample consisted exclusively of college students from Chinese universities, and the external validity of the findings requires further examination. At the same time, differences in AI adoption may exist across countries or regions due to variations in the level of AI development, educational policies, and cultural values. Future research could validate the profile structure and path differences across different countries or educational systems. Third, this study only examined the mediating roles of PA and GM and did not include other potential mediating or moderating variables. Future research could further expand the model to explore additional psychological and social mechanisms. Fourth, all data were based on self-reports, which may be subject to social desirability bias or common method bias. Although statistical tests indicated that common method bias was not severe, future studies could incorporate multi-source data (e.g., peer evaluations, behavioral logs, and objective usage records) to enhance the robustness of the findings. Fifth, this study employed a convenience sampling method through an online survey platform, which may limit the representativeness of the sample and introduce selection bias. Although efforts were made to recruit participants from three provinces in China, the non-probability sampling method implies that the findings may not be fully generalizable to the broader population of college students. Future studies should consider using probability sampling strategies (e.g., stratified random sampling) to improve sample representativeness and external validity. Furthermore, this study did not collect data on participants’ prior experience with AI tools (e.g., ChatGPT, DeepSeek, Gemini) or their frequency of AI usage. Given that AI acceptance is likely influenced by one’s familiarity and hands-on experience with AI technologies, the absence of such information limits our ability to control for these potential confounds or examine their moderating effects. Similarly, data on participants’ academic disciplines and institutional types were not collected, precluding subgroup comparisons that could reveal discipline-specific or institution-specific patterns. Future research should incorporate these variables to enhance the precision of the findings and enable more nuanced analyses. Finally, in this study, the effect sizes of the AIL → PA → AIA pathway and the chain mediation pathway accounted for only 6.26 and 4.75% of the total effect, respectively, indicating relatively limited contributions. Future research should further explore other potential mediating pathways or moderating factors.

## 6 Conclusion

Based on affordance theory and implicit theory of intelligence, this study constructed and tested a chain mediation model in which AIL influences college students’ AIA through PA and GM, and further employed latent profile analysis to identify the heterogeneous types of AIL and their differences in mediation pathways. The results indicate that AIL was significantly and positively associated with college students’ AIA, with PA and GM playing both independent and chain mediating roles, among which the mediating effect of GM accounts for the largest proportion (16.63%). In addition, three heterogeneous latent classes of AIL among college students were identified, namely low AIL–moderate ethics, moderate AIL–low ethics, and high-level balanced, and significant differences were observed across these groups in the chain mediation pathways. Overall, compared with single-dimension-dominant patterns of AIL, a comprehensive cultivation of AIL was associated with higher PA, stronger GM, and more positive AI acceptance and sustainable use. These findings provide empirical evidence for the role of AIL in promoting AIA among college students, and suggest potential implications for differentiated AI education and precision-oriented interventions in higher education.

## Data Availability

The original contributions presented in the study are included in the article/supplementary material, further inquiries can be directed to the corresponding authors.
