# Supplementary material for: Latent profiles of college students’ AI literacy and their association with AI acceptance: the sequential indirect role of perceived affordances and growth mindset
Source: Front Psychol. 2026 Aug 19;17:1926324. doi: 10.3389/fpsyg.2026.1926324 (PMC13533741; doi:10.3389/fpsyg.2026.1926324)
Supplement: Supplementary file 1 [file Table_1.pdf]

Table 1 presents the AVE of the PA scale was 0.50 and the CR was 0.80, both meeting the conventional thresholds (AVE ⩾ 0.50, CR ⩾ 0.70) for adequate convergent validity(Fornell and Larcker, 1981).

Table 1. AVE and CR Results for the PA Scale

Factor	AVE	CR
Factor1	0.50	0.80

Note: AVE = Average Variance Extracted, CR = Composite Reliability.

Table 2 presents the AVE of the GM scale was 0.54, exceeding the recommended threshold of 0.50, and the CR was 0.84, exceeding the recommended threshold of 0.70, indicating good convergent validity.

Table 2. AVE and CR Results for the GM Scale

Factor	AVE	CR
Factor1	0.54	0.84

As shown in Table 3, the AVE of each dimension of the AIA scale ranged from 0.49 to 0.58, and the CR of each dimension ranged from 0.73 to 0.84, all exceeding the recommended threshold of 0.70. Although the AVE of Factor4 (0.49) was marginally below the 0.50 threshold, it was very close to the cutoff and the CR of this dimension reached 0.83, well above 0.70. Taken together, the convergent validity of the AIA scale was considered to be at an acceptable level.

Table 3. AVE and CR Results for the AIA Scale

Factor	AVE	CR
Factor1	0.53	0.84
Factor2	0.58	0.82
Factor3	0.50	0.73
Factor4	0.49	0.83

As shown in Table 4, the AVE of each dimension of the AIL scale ranged from 0.51 to 0.65, all exceeding the recommended threshold of 0.50, and the CR of each dimension ranged from 0.79 to 0.85, all exceeding the recommended threshold of 0.70, indicating good convergent validity.

Table 4. AVE and CR Results for the AIL Scale

Factor	AVE	CR
Factor1	0.65	0.85
Factor2	0.51	0.81
Factor3	0.57	0.79
Factor4	0.59	0.82

As shown in Table 5, all HTMT values among the four dimensions of the AIL scale ranged from 0.208 to 0.470, well below the recommended threshold of 0.85(Henseler et al., 2015), indicating good discriminant validity among the four dimensions.

Table 5. Heterotrait-Monotrait Ratio (HTMT) Results for the AIL Scale

	Factor1	Factor2	Factor3	Factor4
Factor1	-			
Factor2	0.429	-		
Factor3	0.366	0.470	-	
Factor4	0.208	0.394	0.387	-

As shown in Table 6, all HTMT values among the four dimensions of the AIA scale ranged from 0.410 to 0.759, all below the recommended threshold of 0.85. The highest value (0.759, between Factor2 and Factor3) was still clearly below the cutoff, indicating good discriminant validity among the four dimensions.

Table 6. Heterotrait-Monotrait Ratio (HTMT) Results for the AIA Scale

	Factor1	Factor2	Factor3	Factor4
Factor1	-			
Factor2	0.482	-		
Factor3	0.553	0.759	-	
Factor4	0.410	0.419	0.534	-

As shown in Table 7, all HTMT values among the four constructs ranged from 0.464 to 0.725, well below the recommended threshold of 0.85, indicating satisfactory discriminant validity among AIL, PA, GM, and AIA.

Table 7. Heterotrait-Monotrait Ratio (HTMT) Results for All Constructs

	AIA	AIL	GM	PA
AIA	-			
AIL	0.725	-		
GM	0.620	0.541	-	
PA	0.500	0.608	0.464	-

Table 8 presents the CFA results for the four-item PA Scale, reporting Coef, λ, SE, 95% CI, and p for each item. Item 1 was fixed at 1.00 as the reference indicator and therefore has no associated SE, CI, or p. For the remaining three items, Coef ranged from 0.74 to 1.10, all SE were below 0.05, and all 95%CI excluded zero, indicating precise and statistically significant estimates. *λ* ranged from 0.65 to 0.76, all exceeding the 0.50 threshold, and all items were significant at $P < 0.001$. These results indicate that the scale maintains acceptable convergent validity despite the elevated RMSEA.

Table 8. Unstandardized and standardized factor loadings of the PA Scale

**Item**	**Coef**	**SE**	**95%CI**	**P**	**λ**
Item 1	1.00	-	-	-	0.73
Item 2	1.10	0.05	[0.95, 1.16]	< 0.001	0.76
Item 3	0.74	0.04	[0.66, 0.83]	< 0.001	0.67
Item 4	0.75	0.04	[0.67, 0.84]	< 0.001	0.65

Note: Coef = unstandardized factor loading (estimated coefficient); SE = standard error of the unstandardized estimate; 95% CI = 95% confidence interval for the unstandardized estimate, computed as Coef ± 1.96×SE; P = significance level; λ = standardized factor loading. Item 1 was specified as the reference indicator (fixed at 1.00) to scale the latent variable; thus no SE, CI, or P value is reported for this item.

Table 9 presents the average latent class probabilities for individuals assigned to each profile. Values on the diagonal represent the mean probability that individuals classified into a given profile actually belong to that profile. For example, the value of 0.935 for C1 indicates that, on average, individuals assigned to the "low AIL–moderate ethics" group had a 93.5% probability of membership in this class. All diagonal values exceeded 0.92, well above the recommended threshold of 0.80, indicating that the three profiles were clearly differentiated and that classification quality was high. Off-diagonal values were consistently low (all below 0.05), further confirming minimal classification ambiguity across profiles.

Table 9. Average Latent Class Probabilities for Most Likely Class Membership

Assigned Class	C1	C2	C3
C1(Low AIL–Moderate Ethics)	**0.94**	0.03	0.04
C2(Moderate AIL–Low Ethics)	0.04	**0.92**	0.04
C3(High-Level Balanced)	0.02	0.01	**0.97**

Note: Diagonal values (bold) represent the mean probability that individuals assigned to a given class actually belong to that class. Values above 0.80 indicate good classification quality.

As shown in Table 10, the three-profile solution converged to an identical log-likelihood value (−36,382.936) across four independent random start seeds, confirming the stability of the selected LPA solution.

Table 10. Final Stage Log-Likelihood Convergence Across Random Start Seeds (3-Class Model)

Seed	Log-Likelihood
533738	-36,382.936
195873	-36,382.936
68985	-36,382.936
573096	-36,382.936

Note: Convergence to an identical log-likelihood value from four independent random start seeds confirms the stability of the three-profile solution.

To further examine common method bias, a confirmatory factor analysis was conducted by loading all items onto a single common method factor. The results showed that the one-factor model yielded poor fit indices (GFI = 0.60, NFI = 0.44, CFI = 0.48), substantially below the acceptable thresholds, indicating that a single common method factor could not adequately account for the data structure. In contrast, the individual factor models for each scale demonstrated good fit (AIL: GFI = 0.91, CFI = 0.91; AIA: GFI = 0.94, CFI = 0.93; PA: GFI = 0.98, CFI = 0.97;

GM: GFI = 0.98, CFI = 0.98). Taken together with the Harman single-factor test (first factor explaining 22.191% of the total variance, well below the 40% threshold), the converging evidence from both approaches suggests that common method bias is not a serious concern in this study (see Table 11).

Table 11. Confirmatory Factor Analysis-Based Single-Factor Common Method Bias Test

**Model**	**GFI**	**NFI**	**CFI**
One-factor Model	0.60	0.44	0.48
AIL Scale	0.91	0.89	0.91
AIA Scale	0.94	0.91	0.93
PA Scale	0.98	0.97	0.97
GM Scale	0.98	0.97	0.98

References

Fornell, C., and Larcker, D. F. (1981). Evaluating Structural Equation Models with Unobservable Variables and Measurement Error. *Journal of Marketing Research* 18, 39. doi: 10.2307/3151312

Henseler, J., Ringle, C. M., and Sarstedt, M. (2015). A new criterion for assessing discriminant validity in variance-based structural equation modeling. *Journal of the academy of marketing science* 43, 115–135. doi: 10.1007/s11747-014-0403-8
